# Freud's Dream Interpretation: A Different Perspective Based on the Self-Organization Theory of Dreaming

**DOI:** 10.3389/fpsyg.2018.01553

**Published:** 2018-08-23

**Authors:** Wei Zhang, Benyu Guo

**Affiliations:** Research Institute of Moral Education/School of Psychology, Nanjing Normal University, Nanjing, China

**Keywords:** dream interpretation, self-organization, memory consolidation, dream-work, order parameter

The self-organization theory of dreaming proposes that the sleeping brain is a self-organizing system that can combine discontinuous and incongruous neuronal signals (i.e., different elements of dreams) into a relatively continuous narrative during sleep (Kahn and Hobson, 1993; Kahn et al., 2000, 2002). This theory also implies that dreams are not independently functional but rather a coproduct of the sleeping brain, reflecting the dreamer's physiological and psychological activities such as memory consolidation, emotion regulation, and reception of external stimuli (Zhang, 2016).

By contrast, Freud regarded dreams as a royal road to the unconscious; dream interpretation has thus been an important psychoanalytic technique. His theory of dreams mainly refers to two key points: (a) what are the materials of a dream? and (b) how do these materials work together? The answers to these questions are closely related to an understanding of dream interpretation. In this article, we refer to the self-organization theory of dreaming and seek to elucidate its meaning for dream interpretation.

## What are the materials of a dream?

According to Freud (1900), sources of dreams include stimuli from the external world, subjective experiences, organic stimuli within the body, and mental activities during sleep (p. 22). Empirical evidence has supported some of these assertions. The self-organization theory of dreaming posits that memory consolidation, emotion regulation, and reception of external stimuli can contribute to dream content (Zhang, 2016); hence, dream content can contain important information about the dreamer.

Consider, for example, the case of memory consolidation during sleep: according to the two-stage memory model (McClelland et al., 1995; Stickgold and Walker, 2005; Born and Wilhelm, 2012), the process of memory consolidation generates memory fragments to extract pertinent information when an individual is asleep. Moreover, salient memories for the sleeper, such as newly encoded memories (Born and Wilhelm, 2012; Wamsley, 2014), memories that will be incorporated into long-term memory within 6–7 days (e.g., Blagrove et al., 2011; van Rijn et al., 2015), and corresponding long-term memories (Lewis and Durrant, 2011), are preferentially activated and then manifested in dream content. In addition, rapid eye movement (REM) sleep and non-REM (NREM) sleep refer to the processing of different types of memories: REM sleep is primarily implicated in emotional memory and implicit memory, whereas NREM sleep is more closely associated with declarative memory (Rauchs et al., 2005; Smith, 2010). From this perspective, newly encoded memories are related to what Freud (1900) called the “day's residues” in that they reflect some daytime activities of the dreamer. Temporarily stored memories, denoted as the “dream-lag effect,” offer another window into a patient's recent life. Long-term memory is correlated with remote events, implying that dream content may harken back to early experiences (e.g., childhood trauma). Remote memory may even involve information collected over the course of evolution and reflected in typical dream themes, such as flying and being chased (e.g., Revonsuo, 2000; Valli and Revonsuo, 2009; Mathes et al., 2014; Yu, 2016). Moreover, many psychoanalysts have emphasized emotional memories in dream content. Freud (1900) found that affect remains stable in the process of dream formation, at least with respect to quality (p. 460–487). Affect or emotion can be a gateway to learning more about the state of the dreamer. “The principle of affective organization of memory” suggests that the memory network is organized by affect (see Reiser, 2001); accordingly, a therapist may be able to identify a patient's similar affective memories (e.g., traumatic experiences) via emotional material in dream content.

The focus on transference dreams and countertransference dreams in contemporary psychoanalysis aligns with this point. Unconscious communications between the patient and analyst may be reflected in dream content as either the day's residues or a major emotional focus. As such, the therapist can perceive and address interaction issues in treatment (e.g., transference, resistance, countertransference, and counterresistance) based on these dreams (e.g., Hill et al., 2014; Sirois, 2016; Ogden, 2017). To understand and use dreams in this way implies a focus on manifest rather than latent dream content. However, the self-organization theory of dreaming does not endorse the existence of latent dream content (see the following section), although many analysts have expressed interest in manifest dream since the 1950s (see Spanijaard, 1969; Lane, 1997). Even Freud came to realize the significance of such content and suggested considering it seriously at the end of his life (Jiménez, 2012).

Some researchers have contended that manifest dream content represents the whole dream, with no element of distortion or disguise. For example, Kavanagh (1994) advocated eschewing Freudian latent content and instead proposed that manifest content constitutes “real” dreams. Greenberg and Pearlman (1999) proposed that if psychoanalysts understand how manifest dream content attempts to convey the dreamer's problems and resolve them, then a dream can be expressed directly rather than through an obscured purpose. Jennings (2007) purported that dreams are self-evident in that they directly reflect the dreamer's experiences, traits, and wishes. From this perspective, the therapist should use the self-evident method to discover dreams as they truly are in therapy. That is, even if no hidden implications exist, dreams can still be a tool by which the therapist and patient can work together to enrich the therapeutic process. Nevertheless, Freud paid close attention to how these materials combine, and his technique of dream interpretation also relied on this integration, which brings us to our second point.

## How do the materials of a dream work together?

Freud (1900) realized that dream content is derived from but not identical to real life; thus, he suggested that some transformation and connection must exist between these materials. He contended that these connections are not random but rather constrained by one's unconscious desires, such that “a dream is the fulfillment of a wish” (p. 122). He also found that “disagreeable” dreams seem more widespread than “pleasant” dreams (p. 134), hence his hypothesis that dreams can disguise their true purpose (i.e., indirectly fulfilling wishes). Freud therefore identified two types of dreams: manifest dream and latent dream. He stated that the latent dream is the real dream, and the goal of dream interpretation is to reveal it.

To further elaborate on this idea, Freud proposed four mechanisms by which latent dream can be obscured. *Condensation* refers to the reduction and simplification of rich contents of latent dream. *Displacement* refers to a process that substitutes various aspects (e.g., constituents, intensity, significance, and properties) of manifest and latent dream to render them dissimilar. *Symbols* indicate that latent dream is expressed by relevant signs. *Secondary revision* involves making disordered and incoherent dream materials more well-organized and reasonable. This mechanism causes a dream to appear meaningful, but the presented dream is in fact quite different from its actual implication (Freud, 1900).

The self-organization theory of dreaming does not concur with this viewpoint; instead, it offers a different perspective on Freud's dream-work. According to this theory, the nature of condensation is the fragmentation of memories during consolidation, as this process must extract important information for further processing. Displacement, from this perspective, depends on weak control of the sleeping brain and the high degree of freedom in dream elements. In this state, the brain develops new connections between different elements; then, the self-organization mechanism combines various elements to construct a relatively concordant “story,” deemed *secondary revision* per Freud's theory. No analogous symbols exist in dream content because the elements presented therein are merely memory fragments and other components of information processing (see Zhang, 2016). Some researchers have also pointed out that dream symbols are too far-fetched (see Freud, 1916). In other words, dreams are not riddles to be translated (Hartmann, 2010a), and “the manifest dream is the real dream” (Jiménez, 2012); thus, Freud's dream-work does not exist according to this point of view.

However, this position does not mean that the self-organization mechanism does not provide support for dream interpretation. According to self-organization theory, the sleeping brain's control of physical and mental activities is weaker than in the awake brain (Kahn et al., 2000, 2002). Thus, dream elements are characterized by more freedom than mental content when an individual is awake, rendering hyperassociativity between these components possible (Hartmann, 2010b; Horton and Malinowski, 2015). This is why dreams can present bizarre scenes and “miracles” that cannot happen in real life (Zhang, 2016). Nevertheless, this “disordered state” is not unlimited; it could impair brain function otherwise. Therefore, the self-organization mechanism is necessary: it provides a relatively stable state for the dreaming brain, as it can offer a way in which the system can give rise to ordered behavior, structure, or pattern from disorder (Haken, 1977; Prigogine and Stengers, 1984; Fingelkurts et al., 2013). Order parameters or collective variables are crucial during this process.

A system's state is generally determined based on many variables that describe its different aspects. Under certain conditions, the interactions between different components will cause the system to reach a critical state at which most of these variables vanish quickly. To put it another way, few variables survive, but those that do reflect the state of this system. These variables (i.e., order parameters or collective variables) also serve as an invisible hand that controls the operation and evolution of the system, leading to the emergence of a new ordered pattern or behavior. The formation of order parameters is therefore the key channel through which self-organization can successfully continue (see Haken, 1977; Fingelkurts et al., 2013).

Emotion is a pivotal factor during sleep. Several investigations have revealed that dreams are often accompanied by emotions, especially negative ones (e.g., Valli et al., 2008; Malinowski and Horton, 2014). Many researchers have identified a close relationship between dreams and emotions (e.g., Desseilles et al., 2011), even labeling emotions indispensable to dream formation. For instance, Reiser (1997) noted that images serving as nodal points in an individual's memory network are connected by similar types of affect, indicating that affect plays an essential role in memory organization. Reiser (1997) further conjectured that strong affect during sleep evokes existing images that are loaded with similar affect and hence activate relevant earlier experiences to form a dream. Clément (2008) hypothesized the following chain of processes: emotions in sleep are activated and combine to form different emotional scripts, which then serve as templates and replicate a series of images to finally construct a dream narrative. Hartmann (2010b) stated that combinations of dream elements are not random but rather guided by emotion; accordingly, dreams are helpful for building and rebuilding an individual's emotional memory system. In brief, emotions likely play the role of order parameters: they control and guide combinations of dream elements. Emotions can therefore serve as a springboard in comprehending dreams. For instance, perhaps traumatic experiences constitute a core theme of a patient's dreams, suggesting an avenue for further treatment.

## Conclusion

The self-organization theory of dreaming offers a framework distinct from psychoanalytic theories to explain how dreams are generated and operate. This theory proposes that dreams are a byproduct of the dreamer's physical and mental state during sleep, distinguishes between manifest and latent dream, and points out that the dream-work proposed by Freud is actually a result of information processing and self-organization in the sleeping brain. However, this theory allows the therapist to derive important information (e.g., significant memories) from dream content and underscores emotions as a potential order parameter that can provide an effective means of grasping the core of a dream. Therefore, dream analysis may still prove useful in the therapeutic process.

## Author contributions

WZ is responsible for the writing of this paper. BG is in charge of the idea.

### Conflict of interest statement

The authors declare that the research was conducted in the absence of any commercial or financial relationships that could be construed as a potential conflict of interest.
